# Comparison of Humoral Antibody Responses and Seroconversion Rates between Two Homologous ChAdOx1 nCoV-19 and mRNA-1273 Vaccination in Patients Undergoing Maintenance Hemodialysis

**DOI:** 10.3390/vaccines11071161

**Published:** 2023-06-27

**Authors:** Shih-Hsin Hsiao, Yuh-Mou Sue, Chih-Chin Kao, Hui-Wen Chang, Yen-Chung Lin, Ching-Sheng Hung, Yi-Chen Hsieh, Shiao-Ya Hong, Chi-Li Chung, Jer-Hwa Chang, Ying-Shih Su, Ming-Che Liu, Kevin Shu-Leung Lai, Ko-Ling Chien, Jude Chu-Chun Wang, Chung-Yi Cheng, Te-Chao Fang

**Affiliations:** 1 Division of Pulmonary Medicine, Department of Internal Medicine, School of Medicine, College of Medicine, Taipei Medical University, Taipei 11031, Taiwan; 2 Division of Pulmonary Medicine, Department of Internal Medicine, Taipei Medical University Hospital, Taipei 11031, Taiwan; 3 Division of Nephrology, Department of Internal Medicine, School of Medicine, College of Medicine, Taipei Medical University, Taipei 11031, Taiwan; 4 Division of Nephrology, Department of Internal Medicine, Wan Fang Hospital, Taipei Medical University, Taipei 11696, Taiwan; 5 Taipei Medical University-Research Center of Urology and Kidney (RCUK), School of Medicine, College of Medicine, Taipei Medical University, Taipei 11031, Taiwan; 6 Division of Nephrology, Department of Internal Medicine, Taipei Medical University Hospital, Taipei Medical University, Taipei 11031, Taiwan; 7 School of Medical Laboratory Science and Biotechnology, College of Medical Science and Technology, Taipei Medical University, Taipei 11031, Taiwan; 8 Department of Laboratory Medicine, Taipei Medical University Hospital, Taipei Medical University, Taipei 11031, Taiwan; 9 Department of Laboratory Medicine, Wan Fang Hospital, Taipei Medical University, Taipei 11031, Taiwan; 10 College of Medical Science and Technology, Taipei Medical University, Taipei 11031, Taiwan; 11 College of Public Health, Taipei Medical University, Taipei 11031, Taiwan; 12 Department of Biotechnology and Laboratory Science in Medicine, National Yang Ming Chiao Tung University, Taipei 11221, Taiwan; 13 School of Respiratory Therapy, College of Medicine, Taipei Medical University, Taipei 11031, Taiwan; 14 Division of Pulmonary Medicine, Department of Internal Medicine, Wan Fang Hospital, Taipei Medical University, Taipei 11696, Taiwan; 15 Division of Infectious Disease, Department of Internal Medicine, Wan Fang Hospital, Taipei Medical University, Taipei 11696, Taiwan; 16 Department of Internal Medicine, School of Medicine, College of Medicine, Taipei Medical University, Taipei 11031, Taiwan; 17 Department of Urology, Taipei Medical University Hospital, Taipei 11031, Taiwan; 18 Clinical Research Center, Taipei Medical University Hospital, Taipei 11031, Taiwan; 19 School of Dental Technology, College of Oral Medicine, Taipei Medical University, Taipei 11031, Taiwan; 20 Department of Critical Care Medicine, Taipei Medical University Hospital, Taipei 11031, Taiwan

**Keywords:** hemodialysis, COVID, vaccine response

## Abstract

Background: Hemodialysis patients are at an increased risk of SARS-CoV-2 infection and are excluded from preauthorization COVID-19 vaccine trials; therefore, their immunogenicity is uncertain. Methods: To compare the antibody responses to homologous ChAdOx1 and mRNA-1273 SARS-CoV-2 vaccination in hemodialysis patients, 103 age- and sex-matched hemodialysis patients with two homologous prime-boost vaccinations were recruited to detect anti-receptor-binding domain (RBD) IgG levels and seroconversion rates (SCRs) 14 days after a prime dose (PD14), before and 28 days after a boost dose (pre-BD0 and BD28). Results: Both mRNA-1273 and ChAdOx1 vaccinations elicited immunogenicity in study subjects, and the former induced higher anti-RBD IgG levels than the latter. The SCRs of both groups increased over time and varied widely from 1.82% to 97.92%, and were significantly different at PD14 and pre-BD0 regardless of different thresholds. At BD28, the SCRs of the ChAdOx1 group and the mRNA-1273 group were comparable using a threshold ≥ 7.1 BAU/mL (93.96% vs. 97.92%) and a threshold ≥ 17 BAU/mL (92.73% vs. 97.92%), respectively, but they were significantly different using a threshold ≥ 20.2% of convalescent serum anti-RBD levels (52.73% vs. 95.83%). The seroconversion (≥20.2% of convalescent level) at BD28 was associated with mRNA-1273 vaccination after being adjusted for age, sex, body mass index, and the presence of solicited reactogenicity after a prime vaccination. Conclusion: Our prospective, observational cohort indicates that a full prime-boost mRNA-1273 vaccination is likely to provide higher immune protection in hemodialysis patients compared to ChAdOx1, and this population with a prime-boost ChAdOx1 vaccination should be prioritized for a third dose.

## 1. Introduction

People with chronic kidney disease (CKD) are at an increased risk of infection. Patients with CKD as well as with other underlying chronic health conditions, including diabetes mellitus, chronic cardiovascular disease, chronic respiratory disease, and severe obesity, are more prone to severe COVID-19 [1,2]. Moreover, patients with end-stage renal disease (ESRD) and COVID-19 are at high risk for hospitalization, a longer stay at the hospital, a shorter time from symptom onset to intensive care unit admission, and COVID-19 related death [3,4], which further burdens healthcare systems. Thus, the prevention of severe airway respiratory syndrome (SARS) CoV-2 infection is an urgent and important issue for patients with either CKD or ESRD during the COVID-19 pandemic.

Patients with CKD or ESRD are generally considered as immunocompromised because CKD can cause premature aging of the immune system. Uremia can further reduce the number and function of lymphoid cells, and the immune dysregulation of T cells is amplified after the start of renal replacement therapy-dialysis [5,6]. Furthermore, ESRD patients tend to exhibit significant reductions in most B lymphocyte cell subpopulations [7] and have a weak immune response to vaccination, including anti-viral vaccines [8,9]. For example, the effectiveness of HBV vaccination was reported to be lower in patients with ESRD than those without ESRD [10], and this population commonly presented lower peak antibody titers and lower seroconversion rates [11]. Consequently, ESRD patients are usually excluded from preauthorization vaccine trials, including COVID-19 candidate vaccine trials.

The coronavirus, also known as SARS-CoV-2, infects people by coming into contact with contaminated surfaces and then touching one’s face, or through respiratory droplets when an infected person coughs or sneezes. The virus enters the body through the nose, mouth, or eyes, and then binds to specific receptors on the surface of human cells, particularly in the respiratory tract. Once the virus gains entry into the cells, it starts replicating itself using the cellular machinery of the host. As the virus replicates, it damages the cells, leading to inflammation and other symptoms such as coughing, fever, and shortness of breath. Vaccines work by training the immune system to recognize and fight the virus without causing disease. There are currently several types of COVID-19 vaccines, including mRNA vaccines (such as Pfizer-BioNTech and Moderna), viral vector vaccines (such as AstraZeneca and Johnson & Johnson), and protein subunit vaccines (such as Novavax). Several COVID-19 vaccines have shown viral efficacy (VE) for protection from symptomatic COVID-19 in subjects who met restricted inclusion and exclusion criteria and were officially approved for emergency use against the COVID-19 pandemic based on their phase 3 clinical trial results [12,13,14,15]. However, it is uncertain whether either COVID-19 vaccine can evoke a robust immune response and provide convincing VE of protection from symptomatic COVID-19 in the ESRD population in the same way as it does in healthy people. The ChAdOx1 nCoV-19 vaccine consists of a replication-deficient chimpanzee adenoviral vector, ChAdOx1, containing the SARS-CoV-2 structural surface glycoprotein antigen (spike protein; nCoV-19) gene. The mRNA-1273 vaccine is a lipid nanoparticle-encapsulated mRNA-based vaccine that encodes the prefusion-stabilized full-length spike protein of SARS-CoV-2. ChAdOx1 nCoV-2 (ChAdOx1) and mRNA-1273 SARS-CoV-2 (mRNA-1273) vaccines are two of the COVID-19 vaccines most commonly officially approved for emergency use worldwide; however, to the best of our knowledge, comparative data about antibody responses and seroconversion rates before and after a boost vaccination in ESRD patients is limited [16]. Notwithstanding, these data remain of interest and are important for public health policy. Without a doubt, cellular and humoral immunities both contribute to some degree of protection. Recent reports have shown that levels of humoral antispike IgG, antireceptor binding domain (anti-RBD) IgG, and neutralizing antibodies evoked by COVID-19 vaccines correlated with the immune protection from symptomatic COVID-19 [17]. Furthermore, neutralizing antibodies evoked are highly predictive of the VE of COVID-19 vaccines [18]. In this study, we aimed to compare the humoral antibody responses and the rates of positive seroconversion that represented ≥ 50% of the VE of immune protection from symptomatic COVID-19 in ESRD patients with maintenance hemodialysis who received a homologous prime (1st dose)-boost (2nd dose) of mRNA-1273 or ChAdOx1 vaccination.

## 2. Methods

### 2.1. Study Approval and Patients

This prospective observational cohort study was conducted at the dialysis centers of two university-affiliated hospitals in Taiwan following the principles of the Declaration of Helsinki and Good Clinical Practice under the approval of Taipei Medical University Joint Institutional Review Board (N202106049). Enrolled participants were as follows: 20–85 years, undergoing maintenance hemodialysis, not pregnant, and scheduled to receive two homologous prime-boost (PB) mRNA-1273 or ChAdOx1 vaccinations according to the local public health policy. Excluded participants were as follows: patients with a history of prior COVID-19 diagnosis or attendance at a COVID-19 vaccine trial, or with a new onset of fever, cough, shortness of breath, or anosmia within 14 days before enrollment, or with an unstable general condition as judged by physicians. Administration of heterologous COVID-19 vaccines was not allowed during the study period according to the local health policy. Between 17 June 2021 and 7 September 2021, a total of 455 patients were eligible; 69 and 386 received a prime mRNA-1273 vaccination at a dose of 100 mcg and a prime ChAdOx1 vaccination at a dose of 5 x 10^10^ viral particles, respectively. After the age- and sex-matching process, 56 patients with a prime mRNA-1273 vaccination (the mRNA-1273 group) and 56 with a prime ChAdOx1 vaccination (the ChAdOx1 group) (ratio 1:1) were initially recruited into the study. Patients who received prime and boost vaccine doses and provided blood samples 28 days after a boost dose (BD28) were considered to be fully vaccinated. Four patients, including one with a prime ChAdOx1 vaccination and 3 with a prime mRNA-1273 vaccination, refused to take a boost, and another two with a prime mRNA-1273 vaccination lost follow-up. Finally, a total of 103 patients (55 receiving ChAdOx1, 48 receiving mRNA-1273) were fully vaccinated and their data were analyzed.

### 2.2. Study Procedure, Primary Endpoints, and Serological Assays

Every patient underwent a screening visit, where inclusion/exclusion criteria and medical history review were assessed. All patients provided written informed consent and were not blinded. The primary endpoints were the humoral antibody responses to a homologous prime-boost vaccination and positive seroconversion rates (SCRs) at 28 days after a boost vaccination (BD28). Peripheral blood samples obtained at different time points, including before and 14 days after a prime dose (PD0, PD14) and before and 28 days after a boost dose (pre-BD0, BD28), underwent serological assays. Thresholds for anti-RBD IgG seroconversion positivity were determined by three definitions: ≥7.1 BAU/mL according to the manufacturers’ guidelines, ≥17 BAU/mL (representing ≥50% of VE of immune protection from original symptomatic COVID-19 in four ChAdOx1 vaccine trials) [18], and ≥20.2% of convalescent serum NT (representing ≥50% of VE of immune protection from original symptomatic COVID-19, estimated in Khoury et al. using pooled analyses of NT and VE data from eight different vector-based SARS-CoV-2 candidate vaccine phases 1–3 preauthorization trials) [17]. Our previous study showed that serological anti-RBD IgG levels evoked by ChAdOx1 vaccination were highly correlated to anti-spike IgG levels and neutralizing antibody titers (NT) measured by a live virus neutralizing assay [19], respectively. In this study, the convalescent anti-RBD IgG levels were tested with the SARS-CoV-2 IgG II assay (Abbott, Sligo, Ireland), where a positive result was defined as ≥50 arbitrary units (AU)/mL and presented as binding affinity units (BAU)/mL (AU/mL × 0.142 can be converted to BAU/mL) and used as a surrogate of convalescent NT when analyzing SCRs. The convalescent serum anti-RBD IgG levels were retrieved from 34 age- and sex-matched laboratory-confirmed COVID-19 patients with different disease severities, including 2 asymptomatic, 18 mild, 2 moderate, 3 severe, and 9 critical illnesses, at post-diagnosis 33–134 days with a median (IQR), 72 (64.25–83.75) days (study number: N202107004), and they were not previously published. The serologic assay for measuring anti-RBD IgG levels of the convalescent serum and the study cohort’s serum was the same and was performed at the same laboratory (Taipei Municipal Wang Fang Hospital, Taipei, Taiwan) accredited by the Taiwan Centers for Disease Control. The solicited reactogenicity within 14 days after a prime dose administration was self-reported through a questionnaire.

### 2.3. Statistics and Analyses

The statistical analysis was performed using SAS 9.4 (Cary, NC, USA) and GraphPad Prism version 9.0 (GraphPad Software, San Diego, CA, USA). Continuous variables are shown as mean with standard deviation (SD) and are examined using Student t test if the distribution is normal, while data were reported as median with interquartile ranges (IQRs) and tested using Mann–Whitney U test when the normal distribution is not followed. Categorical variables are presented as frequencies with percentages and are examined using chi-square test or Fisher exact test when appropriate. Multivariate logistic regression model was used to investigate the association between anti-RBD IgG seroconversion at BD28, the ChAdOx1 group, and the mRNA-1273 group after adjustment for age, sex, body mass index (BMI), and the presence of solicited reactogenicity after a prime COVID-19 vaccination.

## 3. Results

### 3.1. Baseline Characteristics of Study Population

The mean ages of the ChAdOx1 group and the mRNA-1273 group were 64.24 (SD 11.66) years and 63.67 (SD 12.52), respectively (*p* = 0.8116), and females accounted for 50.91% of the former and 50% of the latter, respectively (*p* = 0.9267) (Table 1). The mean time of dialysis history was similar in both groups (4.37 [SD 5.2] years for the ChAdOx1 group vs. 5.54 [SD 5.46] years for the mRAN-1273 group, *p* = 0.1810). There was no statistical difference between the two groups regarding baseline body mass index (BMI), white blood cell count, hemoglobin level, the PB interval, or the time of serological tests after a booster dose.

### 3.2. Humoral Antibody Responses Induced by ChAdOx1 and mRNA-1273 Vaccination

**Figure 1:**
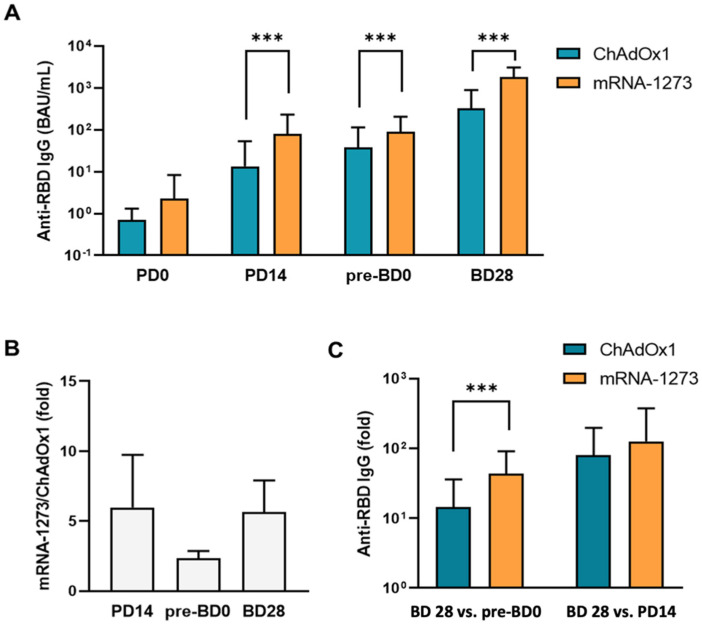
Antibody responses in ChAdOx1, nCoV-19, and mRNA-1273-vaccinated hemodialysis patients. (**A**) Time-dependent changes of anti-RBD IgG levels induced by ChAdOx1, nCoV-19, and mRNA-1273 vaccination. (**B**) Comparison of mean anti-RBD IgG levels induced by different vaccines. (**C**) Comparison of mean anti-RBD IgG levels induced by vaccination at various time points. *** denotes *p* < 0.001.

**Figure 2:**
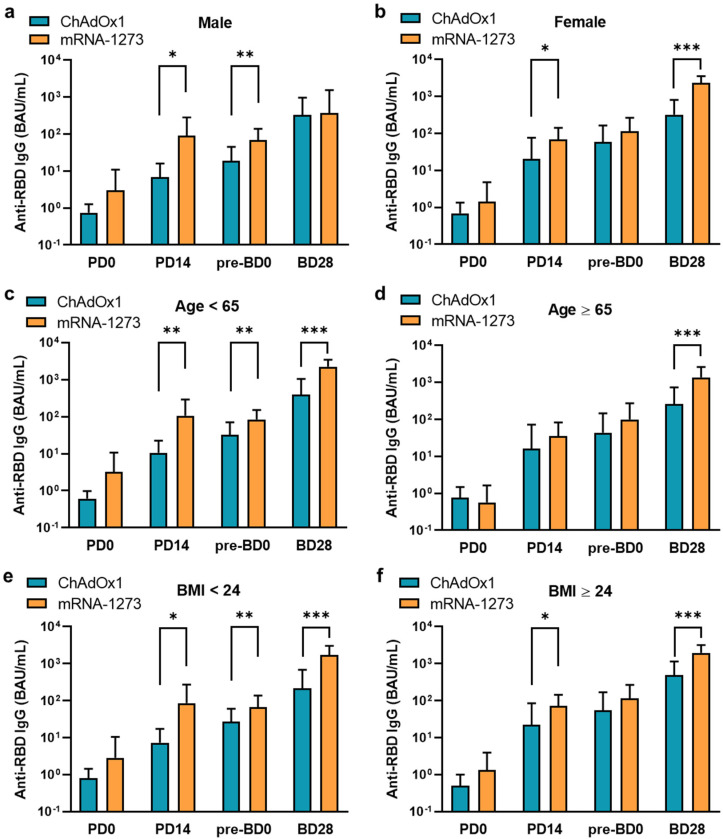
Comparison of vaccine-induced anti-RBD IgG levels at different time points and in different subgroups. (**a**,**b**) Antibody responses in different genders. (**c**,**d**) Antibody responses at different ages. (**e**,**f**) Antibody responses in different BMIs. *, **, and *** denote *p* < 0.05, <0.01, and <0.001.

The mean anti-RBD IgG level induced by ChAdOx1 vaccination increased over time and was 13.37 BAU/mL (SD 40.1) at PD14, 38.13 (SD 76.71) at pre-BD0, and 322.95 [SD 562.35] at BD28 (Figure 1A and Table S1 in the Supplementary data), and that induced by mRNA-1273 SARS-CoV-2 vaccination was 79.91 BAU/mL (SD 150.8) at PD14, 89.78 (SD 116.68) at pre-BD0, and 1822.88 (SD 1276.45) at BD28, which were significantly higher when compared to that induced by ChAdOx1 vaccination at different time points (trend *p* < 0.0001). The mean anti-RBD IgG level induced by mRNA-1273 vaccination was 5.98 (SD 3.76) fold higher than that induced by ChAdOx1 one at PD14, 2.35 (SD 0.52) at pre-BD0, and 5.64 (SD 2.27) at BD28, respectively (Figure 1B). The mean anti-RBD IgG level induced by mRNA vaccination at BD28 was 43.73 fold (SD 47.30) of that at pre-BD0; and the mean anti-RBD IgG level induced by ChAdOx1 at BD28 was 14.44 (SD 21.81) fold of that at pre-BD0 (Figure 1C). Further subgroup analyses showed similar results; the mRNA COVID-19 vaccination induced higher anti-RBD IgG levels at different time points and in different subgroups when compared to ChAdOx1 vaccination (Figure 2, Table S2, Figures S1 and S2 in the Supplementary data).

### 3.3. Seroconversion Rates between ChAdOx1 and mRNA-1273 Vaccination

The anti-RBD IgG SCRs increased over time (e.g., 30.91% at PD14, 73.08% at pre-BD0, and 96.36% at BD28 for the ChAdOx1 group by a cut-off threshold of 7.1 BAU/mL) and varied widely in terms of different thresholds of positive seroconversion (Table 2). The SCRs at PD14 and at pre-BD0 were significantly different between the two groups regardless of the different thresholds (e.g., 18.18% vs. 60.57%, *p* < 0.0001 at PD14; 53.85% vs. 85.49%, *p* = 0.0073 at pre-BD0 using a threshold of ≥17 BAU/mL). The SCRs at BD28 of the ChAdOx1 group and the mRNA-1273 group were comparable either using a threshold of ≥ 7.1 BAU/mL (96.36% vs. 97.92%, *p* > 0.999) or using a threshold of ≥ 17 BAU/mL (92.73% vs. 97.92%, *p* = 0.3686), but they were significantly different using a threshold of ≥ 20.2% of convalescent serum anti-RBD IgG levels (52.73% vs. 95.83%, *p* < 0.0001).

Multivariate logistic regression model analyses were used to elucidate which variable was associated with the positive anti-RBD IgG seroconversion at BD28 in hemodialysis patients with a prime-boost vaccination and found that no variable was significantly associated with positive seroconversion using either a threshold of ≥7.1 BAU/mL or ≥17 BAU/mL (Table 3). When the threshold was set at 20.2% of convalescent serum anti-RBD IgG levels, the administration of mRNA-1273 vaccine and BMI ≥ 24 were independently and significantly associated with positive seroconversion, respectively (OR = 30.88, 95% CI 6.04–157.96, *p* < 0.0001 and OR = 4.35, 95% CI 1.32–14.37, *p* = 0.0160, respectively) (Table 3). The hemodialysis patients with any of the solicited reactogenicities (fatigue, tenderness, fever, and headache) after a prime vaccination had a trend towards positive seroconversion (OR = 3.44, 95% CI 0.99–11.93, *p* = 0.0519). The presence of diabetes mellitus or hypertension was not included in this multivariate logistic regression analysis because of the small patient number.

## 4. Discussion

This prospective observational study with an age- and gender-matched design showed that, although both the mRNA-1273 and ChAdOx1 COVID-19 vaccines were immunogenic and successfully evoked humoral anti-RBD IgG responses over time, the mean anti-RBD IgG levels induced by the former were significantly higher than the latter across different time points in adult patients undergoing maintenance hemodialysis. The pre- and post-boost vaccination SCRs varied widely depending on the definition of cut-off thresholds as well as the vaccine vector types, providing further understanding and insight into humoral antibody responses induced by a PB COVID-19 vaccination in this immunocompromised population.

There are no immunogenic data from patients with maintenance hemodialysis from preauthorization vaccine trials; thus, those from post-authorization observational studies are very important. To reduce the potential bias, the sex and age of the vaccine recipients were matched between the mRNA and the ChAdOx1 groups in this study because prior studies reported that females generated higher antibody responses to mRNA-based COVID-19 vaccines than males [20], and elderly patients with CKD or ESRD had lower antibody responses to COVID-19 vaccine than young patients [21,22]. Little post-authorization research focusing on the comparison of mRNA- and nonreplicating adenovirus-vectored COVID-19 vaccination in ESRD patients has been reported [22], and one study showed that the two-dose BNT162b2 vaccination provided a higher median anti-spike antibody concentration than the two-dose ChAdOx1 in the UK [462 (152-1171) vs. 79 (20-213) BAU/mL, *p* < 0.0001] [23]. This study showed that both mRNA-1273 and ChAdOx1 vaccination could induce anti-RBD IgG responses in ESRD patients with dialysis, and the former evoked much higher antibody responses across different time points than the latter (e.g., 1547.79 [SD 2523.90] vs. 111.21 [SD 255.63] BAU/mL at BD28, *p* < 0.0001) in East Asian patients, which suggested that mRNA-1273 vaccination potentially provided higher immune protection from COVID-19 for this immunocompromised population than the ChAdOx1 vaccination.

The true VE of immune protection from symptomatic COVID-19 in the hemodialysis population remains uncertain. Whether ESRD patients were seropositive after vaccination may be a clue as to whether they have immune protection; however, the definition and thresholds of positive seroconversion (or responder) vary across different studies, which limits the original and meta-analyses data interpretation [16] and may cause confusion. For example, the BNT1262b2 vaccine induced significantly higher antibody responses (around 5.85 folds) than the ChAdOx1 vaccine, but their SCRs were reported to be comparable (88.3 vs. 83.5%, *p* = 0.11) [23]. In this study, the anti-RBD IgG RCRs evoked by either mRNA-1273 or ChAdOx1 vaccine were analyzed using three different thresholds and they varied widely from 1.8% to 97.92% (Table 2). The comparative results between mRNA-1273 and ChAdOx1 vaccines could be reported as similar or significantly different, reflecting the importance of defining positive seroconversion (Table 2). From our perspective, whether the threshold of positive seroconversion could correlate to the VE of immune prevention from symptomatic original COVID-19 (e.g, ≥50%) [17,18] is more important and of clinical significance (Table 2 and Table 3). From this point of view, our data supported the notion that a full prime-boost vaccination is necessary for patients with maintenance dialysis and suggested that this population with the previous homologous PB ChAdOx1 vaccination should be prioritized for the 3^rd^ dose administration of COVID-19 vaccine.

## 5. Limitations

There are several limitations to our study. First, the PB intervals of both mRNA-1273 and ChAdOx1 vaccination were longer than those suggested by the manufacturers, which may have biased our results; however, they reflected the reality during the pandemic. Second, humoral anti-spike IgG and NT concentrations as well as cellular immunity were not tested. Third, the impact of drugs administered and the presence of chromic comorbidities (e.g., diabetes mellitus and hypertension) on overall antibody responses was not further elucidated because of the small patient number. The prevalence of people infected by SARS-CoV-2 in Taiwan was low (less than 1% estimated); however, occult prior SARS-CoV-2 infection could not be, theoretically, excluded in this study cohort without a previously reported medical history of COVID-19. In addition, no age- and gender-matched healthy people were recruited as a control group. These weak points, as mentioned above, limit the data interpretation in this study.

## 6. Conclusions

The administration of any emergent use authorization vaccine will probably attenuate COVID-19-related morbidity and mortality, but the optimal deployment strategy for hemodialysis patients who are yet to start a vaccination course or have only received a prime vaccination remains to be determined. Our comparative data suggest that a full PB vaccination, especially mRNA-1273, is likely to offer broader humoral immune protection and that hemodialysis patients who previously received ChAdOx1 vaccination should be prioritized for a third dose.

## Figures and Tables

**Table 1:** Baseline characteristics of hemodialysis patients. Abbreviation: BMI: body mass index; DM: diabetes mellitus; WBC: white blood cell count; Hb: hemoglobin; PB: prime (1st dose)-boost (2nd dose); SD: standard deviation; IQR: interquartile range.

Variable	ChAdOx1 (*n* = 55)		mRNA-1273 (*n* = 48)		*p* Value
Age, mean (SD)	64.24	(11.66)	63.67	(12.52)	0.8116
Sex, *n*(%)					0.9267
Female	28	(50.91)	24	(50.00)	
Male	27	(49.09)	24	(50.00)	
BMI, mean (SD)	24.05	(4.23)	23.71	(2.99)	0.6357
DM					0.2015
No	24	(43.64)	27	(56.25)	
Yes	31	(56.36)	21	(43.75)	
Hypertension					0.1253
No	14	(25.45)	19	(39.58)	
Yes	41	(74.55)	29	(60.42)	
WBC, median (IQR)	5800	(1960)	5600	(2870)	0.4691
Hb, median (IQR)	10.50	(1.20)	10.55	(1.20)	0.3508
Time of dialysis history (year), median (IQR)	4.37	(5.2)	5.54	(5.46)	0.1810
PB interval (week), median (IQR)	10.71	(3.85)	11.50	(0.35)	0.4839
Time of serological tests after a boost dose (week), Median (IQR)	4.00	(0)	4.00	(0.50)	0.7803

**Table 2:** Seroconversion rates of anti-RBD IgG in hemodialysis patients after vaccination. Abbreviation: SCR: seroconversion rates of anti-RBD IgG; PD14: 14 days after the prime vaccination; pre-BD0: before the boost vaccination; BD28: 28 days after the boost vaccination.

Threshold of SCR	PD14			Pre-BD0			BD28		
	ChAdOx1	mRNA-1273	*p* Value	ChAdOx1	mRNA-1273	*p* Value	ChAdOx1	mRNA-1273	*p* Value
≥7.1 BAU/mL	17/55 (30.91%)	27/35 (77.14%)	<0.0001	38/52 (73.08%)	38/41 (92.68%)	0.0151	53/55 (96.36%)	47/48 (97.92%)	>0.999
≥17 BAU/mL	10/55 (18.18%)	24/35 (68.57%)	<0.0001	28/52 (53.85%)	33/41 (80.49%)	0.0073	51/55 (92.73%)	47/48 (97.92%)	0.3686
≥20.2%	1/55 (1.82%)	7/35 (20.00%)	0.0051	2/52 (3.85%)	12/41 (29.27%)	0.0007	29/55 (52.73%)	46/48 (95.83%)	<0.0001

**Table 3:** The association between variables and seroconversion rates at day 28 after a booster vaccination. Abbreviation: BAU: binding affinity domain; OR: odds ratio; CI: confidence interval; BMI: body mass index; ^#^ Vaccine reactogenicity after a prime vaccination was binary based on the presence of any of the following symptoms after a prime dose: fatigue, tenderness, fever, and headache. * *p* value for multivariate logistic regression analyses.

Variable	Threshold of SCR			
	≥17 BAU/mL		≥20.2%	
	OR (CI 95%)	* p Value	OR (95% CI)	* p Value
Vaccine type (mRNA-1273 vs. ChAdOx1)	3.86(0.39–38.41)	0.2488	30.88(6.04–157.96)	<0.0001
Age (<65 vs. ≥65)	3.72(0.38–37.01)	0.2617	2.03(0.68–6.10)	0.2059
Gender (female vs. male)	4.76(0.47–47.81)	0.1849	1.15(0.37–3.58)	0.8052
BMI (≥24 vs. <24)	2.80(0.27–28.84)	0.3868	4.35(1.32–14.37)	0.0160
Vaccine reactogenicity ^#^ (Yes vs. No)	0.67(0.09–4.89)	0.6901	3.44(0.99–11.93)	0.0519

## Data Availability

All data in this study is available in the main text.

## Supplementary Materials

The following supporting information can be downloaded at: https://www.mdpi.com/article/10.3390/vaccines11071161/s1, Figure S1: Serum anti-RBD IgG levels at different time points after vaccination title; Figure S2: Subgroup analyses of serum anti-RBD IgG levels at different time points after vaccination; Table S1: Serum anti-RBD IgG levels at different time points after vaccination; Table S2: Subgroup analyses of serum anti-RBD IgG levels at different time points after vaccination.
