# Supplementary material for: Comparison of Humoral Antibody Responses and Seroconversion Rates between Two Homologous ChAdOx1 nCoV-19 and mRNA-1273 Vaccination in Patients Undergoing Maintenance Hemodialysis
Source: Vaccines (Basel). 2023 Jun 27;11(7):1161. doi: 10.3390/vaccines11071161 (PMC10383458; doi:10.3390/vaccines11071161)
Supplement: Supplementary file 1 [file vaccines-11-01161-s001.zip › vaccines-2356713-supplementary.pdf]

Comparison of Humoral Antibody Responses and Seroconversion Rates between Two Homologous ChAdOx1 nCoV-19 and mRNA-1273 Vaccination in Patients Undergoing Maintenance Hemodialysis

Supplemental Tables

Table S1. Serum anti-RBD IgG levels at different time points after vaccination

Time point	ChAdOx1		mRNA-1273		*P* value
	N	Mean (SD)	N	Mean (SD)	
PD0	32	0.71 (0.61)	35	2.25 (6.08)	.1588
PD14	55	13.37 (40.1)	35	79.91 (150.8)	.0025
Pre-BD0	52	38.13 (76.71)	41	89.78 (116.68)	.0119
BD28	55	322.95 (562.35)	48	1822.88 (1276.45)	<.0001

Abbreviation: PD0: before the prime vaccination; PD14: 14 days after the prime vaccination; pre-BD0: before the boost vaccination; BD28: 28 days after the boost vaccination; SD: standard deviation.

Table S2. Subgroup analyses of serum anti-RBD IgG levels at different time points after vaccination

Variable	ChAdOx1		mRNA-1273		*P* value
	N	Mean (SD)	N	Mean (SD)	
AGE<65					
PD0	12	0.61 (0.36)	22	3.24 (7.51)	.2375
PD14	27	10.62 (11.87)	22	106.08 (183.38)	.0095
Pre-BD0	25	32.08 (38.21)	25	83.98 (65.93)	.0013
BD28	27	389.24 (645.37)	25	2248.29 (1174.89)	<.0001
AGE≥65					
PD0	20	0.76 (0.72)	13	0.57 (1.07)	.2606
PD14	28	16.01 (55.37)	13	35.61 (46.92)	.0142
Pre-BD0	27	43.74 (100.63)	16	98.84 (170.91)	.1106
BD28	28	259.02 (471.98)	23	1360.48 (1243.31)	<.0001
Male					
PD0	10	0.75 (0.52)	18	3.03 (7.87)	.3726
PD14	28	6.8 (9.15)	20	88.72 (191.57)	.0280
Pre-BD0	27	18.73 (26.21)	21	67.91 (70.22)	.0016
BD28	28	327.39 (636.2)	24	372.95 (1164.5)	.8591
Female					
PD0	22	0.69 (0.66)	17	1.43 (3.34)	.3155
PD14	27	20.17 (56.21)	15	68.17 (71.87)	.0212
BD0	25	59.1 (104.29)	20	112.74 (149.63)	.1642
BD28	27	318.35 (486.25)	24	2272.81 (1245.38)	<.0001
BMI < 24					
PD0	22	0.8 (0.64)	21	2.86 (7.58)	.2110
PD14	33	7.26 (10.08)	21	85.13 (188.02)	.0207
Pre-BD0	31	26.68 (33.43)	22	67.31 (69.5)	.0066
BD28	33	212.23 (466.74)	26	1707.87 (1333.37)	<.0001
BMI ≥ 24					
PD0	10	0.49 (0.5)	14	1.34 (2.57)	.3161
PD14	22	22.54 (61.92)	14	72.07 (70.58)	.0335
Pre-BD0	21	55.04 (113.25)	19	115.8 (152.61)	.1583
BD28	22	489.03 (658.08)	22	1958.8 (1222.44)	<.0001
Non-DM					
PD0	17	0.82 (0.67)	20	2.39 (7.28)	.3827
PD14	24	7.6 (9.62)	21	87.11 (186.57)	.0428
Pre-BD0	21	32.17 (40.87)	24	63.89 (70.07)	.0758
BD28	24	437.11 (669.19)	27	1840.79 (1238)	<.0001
DM					

**PD0**	15	0.58 (0.53)	15	2.07 (4.21)	.1847
**PD14**	31	17.84 (52.7)	14	69.1 (75.62)	.0118
**Pre-BD0**	31	42.18 (94.06)	17	126.32 (156.7)	.0242
**BD28**	31	234.56 (455.42)	21	1799.86 (1354.81)	<.0001
**Non-HT**					
**PD0**	2	0.62 (0.31)	15	1.39 (2.61)	.6909
**PD14**	14	8.71 (10.37)	17	53.26 (73.59)	.0329
**Pre-BD0**	13	21.08 (24.49)	16	60.71 (70.96)	.0658
**BD28**	14	149.43 (156.9)	19	1720.26 (1364.69)	.0002
**HT**					
**PD0**	30	0.71 (0.63)	20	2.9 (7.75)	.1282
**PD14**	41	14.96 (46.11)	18	105.08 (197.5)	.0073
**Pre-BD0**	39	43.82 (87.03)	25	108.38 (136.46)	.0240
**BD28**	41	382.2 (636.23)	29	1890.11 (1235.26)	<.0001

Abbreviation: PD0: before the prime vaccination; PD14: 14 days after the prime vaccination; pre-BD0: before the boost vaccination; BD28: 28 days after the boost vaccination; BMI: body mass index; DM: diabetes mellitus; HT: hypertension; SD: standard deviation.

Supplemental Figures

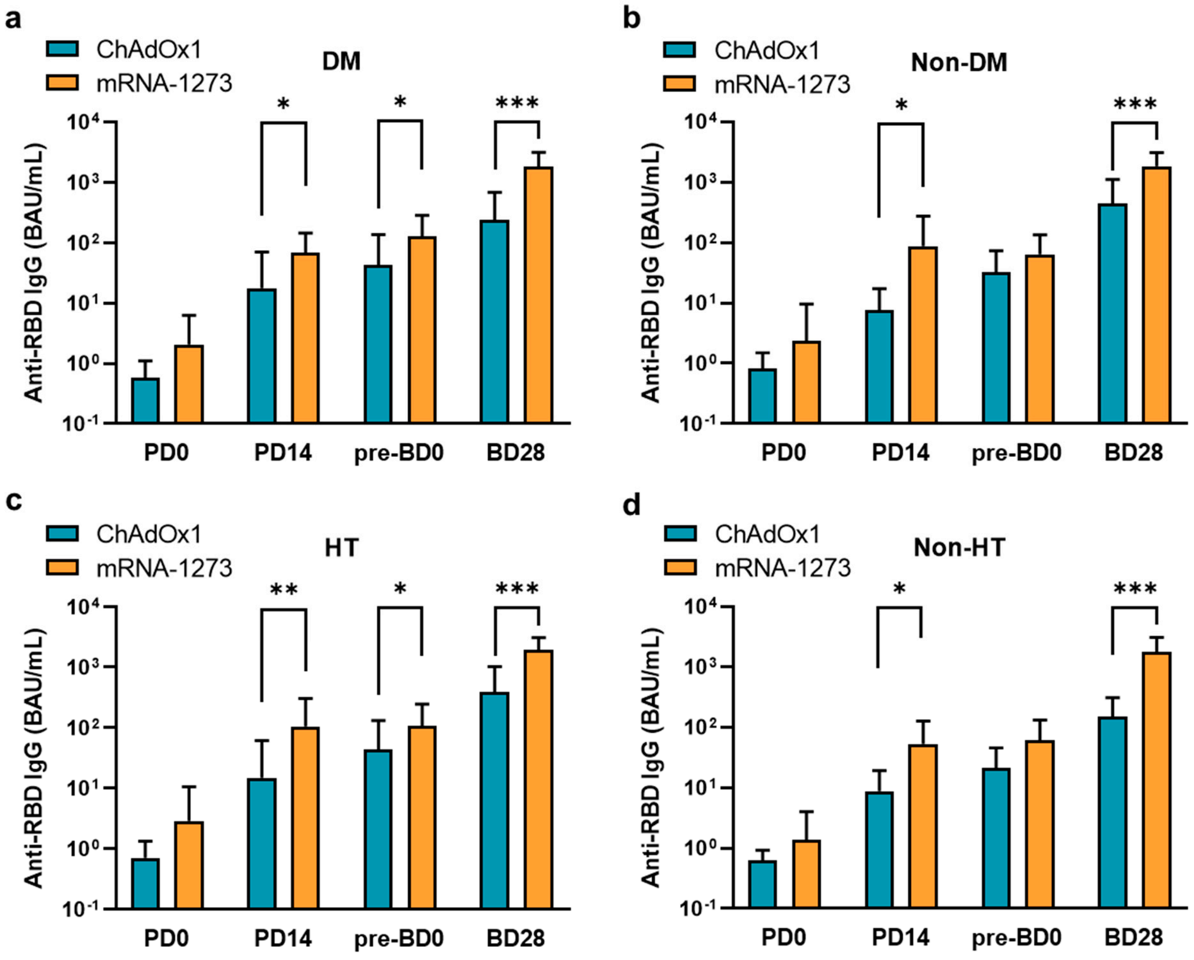

Figure S1. Comparison of vaccine-induced anti-RBD IgG levels at different time points and in different subgroups with or without chromic comorbidities. (a-b) Antibody responses in different subgroups with or without diabetes mellitus (DM). (c-d) Antibody responses in different subgroups with or without hypertension (HT).

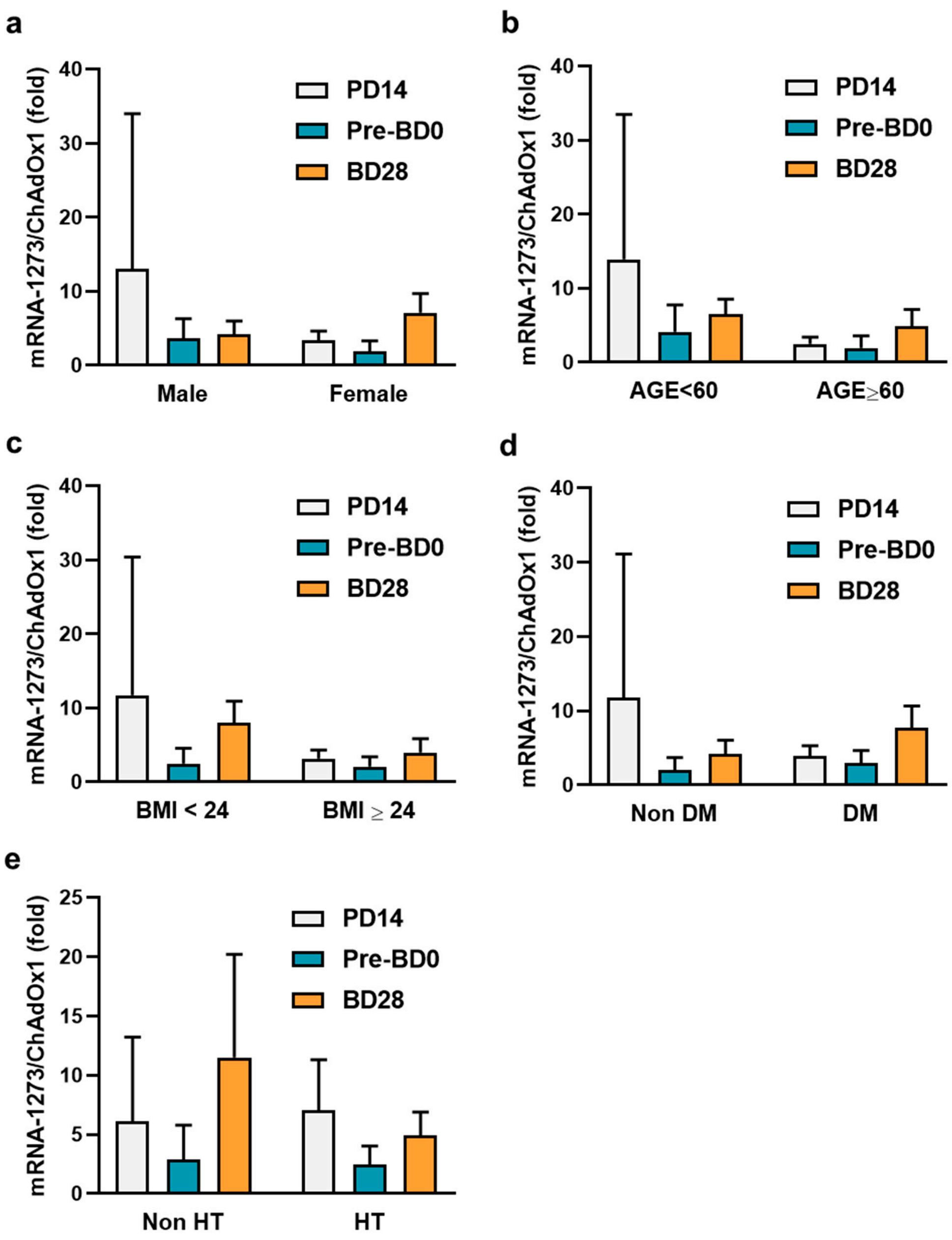

Figure S2. Comparison of anti-RBD IgG levels induced by different vaccines at each time point and in each subgroup. The effect of gender (a), age (b), BMI (c), diabetes mellitus (d) and hypertension (e) on antibody responses induced by different vaccines.
